# Caregiver’s Relationship Type and Race/Ethnic Group Comparisons in a Community-based Caregiver Support Program

**DOI:** 10.1093/geroni/igaa057.3364

**Published:** 2020-12-16

**Authors:** Jinmyoung Cho, Donald Smith, Alan Stevens

**Affiliations:** 1 Baylor Scott & White Health, Temple, Texas, United States; 2 United Way’s Area Agency on Aging of Tarrant County, Fort Worth, Texas, United States

## Abstract

In the past decades, a number of evidence-based interventions has been implemented to improve caregiver outcomes; however, limited attention to specific subgroups (e.g., racial/ethnic groups) still exists in dementia caregiver support interventions in community settings. The purpose of this study is to examine the effect of caregiver’s race/ethnicity and relationship type to the care-recipient on the quality of life among caregivers. This study included 354 informal caregivers enrolled in REACH-TX, a community-based caregiver program, provided by the Alzheimer’s Association North Central Texas Chapter’s from November 2011 to June 2019. Propensity score matching extracted a subset of participants who enrolled in REACH-TX to balance caregivers from three race/ethnic groups (White: 171; African American: 103; Hispanic: 80). Five dimensions of quality of life (depression, caregiving burden, social support, self-care, and problem behaviors) were assessed at baseline and 6-month follow-up among spousal and adult-child caregivers by race/ethnic group. Generalized linear models showed that significant improvement on the five domains of quality of life among White child caregivers, whereas neither spousal nor child caregivers in African American group showed improvement on any domains after adjusting covariates (age, gender, risk levels, number of therapeutic contacts), These findings indicate that the responses to intervention components vary by race/ethnic group and relationship type. Advancing tailored dementia caregiver interventions to specific subgroup needs and unique context, especially for non-White caregivers, is needed to maximize the benefit of community resources and support for diverse caregivers.

